# Preoperative and postoperative prognostic factors of patients with stage II/III lower rectal cancer without neoadjuvant therapy in the clinical trial (JCOG0212)

**DOI:** 10.1093/jjco/hyab183

**Published:** 2021-12-02

**Authors:** Masayuki Ohue, Shin Fujita, Junki Mizusawa, Yukihide Kanemitsu, Tetsuya Hamaguchi, Shunsuke Tsukamoto, Shingo Noura, Masayoshi Yasui, Masaaki Itoh, Akio Shiomi, Koji Komori, Jun Watanabe, Yoshihiro Akazai, Manabu Shiozawa, Takashi Yamaguchi, Hiroyuki Bandou, Kenji Katsumata, Yoshihiro Moriya

**Affiliations:** Department of Gastroenterological Surgery, Osaka International Cancer Institute, Osaka, Japan; Department of Surgery, Tochigi Cancer Center, Tochigi, Japan; Japan Clinical Oncology Group Data Center/Operations Office, National Cancer Center Hospital, Tokyo, Japan; Department of Colorectal Surgery, National Cancer Center Hospital, Tokyo, Japan; Department of Gastroenterological Oncology, International Medical Center, Saitama Medical University, Saitama, Japan; Department of Colorectal Surgery, National Cancer Center Hospital, Tokyo, Japan; Department of Gastroenterological Surgery, Toyonaka Municipal Hospital, Osaka, Japan; Department of Gastroenterological Surgery, Osaka International Cancer Institute, Osaka, Japan; Department of Colorectal Surgery, National Cancer Center Hospital East, Kashiwa, Japan; Division of Colon and Rectal Surgery, Shizuoka Cancer Center Hospital, Shizuoka, Japan; Department of Gastroenterological Surgery, Aichi Cancer Center Hospital, Nagoya, Japan; Department of Surgery, Gastroenterological Center, Yokohama City University Medical Center, Kanagawa, Japan; Department of Surgery, Okayama Saiseikai General Hospital, Okayama, Japan; Department of Surgery, Kanagawa Cancer Center, Yokohama, Japan; Department of Surgery, Kyoto Medical Center, Kyoto, Japan; Department of Surgery, Ishikawa Prefectural Central Hospital, Ishikawa, Japan; Department of Gastrointestinal and Pediatric Surgery, Tokyo Medical University Hospital, Tokyo, Japan; Department of Surgery, Miki Hospital, Iwate, Japan

**Keywords:** rectal cancer, prognostic factor, preoperative, postoperative, survival

## Abstract

**Background:**

The JCOG0212 trial was a randomized controlled trial comparing mesorectal excision alone to mesorectal excision with lateral lymph node dissection for stage II/III lower rectal cancer patients without clinical lateral lymph node enlargement. This study aimed to identify clinicopathological prognostic factors for relapse-free survival and overall survival of lower rectal cancer in the trial.

**Methods:**

Prospective data were selected from 663 patients with complete data. Uni and multivariable Cox regression model was applied to evaluate the preoperative and the combined preoperative and postoperative factors, respectively. Preoperative factors included age, sex, performance status, clinical T, clinical N and operative procedures. Postoperative factors included histological grade, pathological T, number of metastatic lymph nodes and number of dissected lymph nodes. No patient received neoadjuvant treatment.

**Results:**

Regarding preoperative factors, multivariable analysis revealed that performance status 1 (vs. 0: HR 2.079, *P* = 0.0041) and cT4a (vs. cT2–3: HR 2.721, *P* = 0.0002) were independent risk factors for relapse-free survival, and those for overall survival were male (vs. female: HR 1.660, *P* = 0.0228) and cT4a (vs. cT2–3: HR 2.486, *P* = 0.0473). The only independent preoperative risk factor common for relapse-free survival and overall survival was cT4a. Taking preoperative and postoperative factors together, the number of metastatic lymph nodes was the only independent risk factor common for relapse-free survival and overall survival.

**Conclusions:**

Clinical stage II/III lower rectal cancer patients with cT4a should be a target of therapeutic development of neoadjuvant therapy. Postoperatively, intensive chemotherapy should be investigated for patients with more metastatic lymph nodes.

## Teaser

This paper prospectively examined preoperative and postoperative prognostic factors for relapse-free survival and overall survival of patients with stage II/III lower rectal cancer in the JCOG0212 trial.

## Introduction

In Western countries, mesorectal excision (ME) with preoperative chemoradiation is the standard procedure for locally advanced rectal cancer (1). In contrast, ME with lateral lymph node dissection (LLND) is the standard procedure for clinical stage II or III lower rectal cancer in Japan (2). The Japan Clinical Oncology Group (JCOG) 0212 trial (ClinicalTrials.gov: NCT00190541, UMIN-CTR: C000000034) aimed to confirm the non-inferiority of ME alone compared to ME with LLND. The primary endpoint was relapse-free survival and a secondary endpoint was overall survival (3). Primary data and long-term follow-up data did not support the non-inferiority of ME alone in comparison with ME with LLND on the intent-to-treat analysis (3,4). Recurrences were observed in ~30% of patients in the trial. Because neoadjuvant treatment is a possible option to improve relapse-free survival (RFS) and overall survival (OS) for lower rectal cancer, there is an urgent need to identify preoperative prognostic factors to provide optimal treatment for individual patients. Moreover, more intensive adjuvant chemotherapy may be necessary for some postoperative patients, for whom pre- and post-operative prognostic factors should be identified.

Many papers have investigated risk factors, including LLND, for rectal cancer survival without preoperative treatment, however, most of them were based on retrospective data (5,6). Therefore, the present prospective analysis for the preoperative and postoperative factors of lower rectal cancer patients would be of clinical value without much retrospective selection bias. One of the main purposes was to identify which patient-, tumor- and surgery-related characteristics were risk factors for RFS and OS in the JCOG0212 trial. The other was to elucidate preoperative prognostic factors to perform neoadjuvant therapy.

## Materials and methods

The JCOG0212 trial was a multicenter, open-label, randomized, phase III study. The institutional review boards of participating institutions approved the study protocol. Details of the trial have been reported previously (3,4). Operative methods and pathology results were recorded according to the fifth edition of TNM classification (7) and the sixth edition of Japanese Classification of Colon and Rectal Carcinoma (8). Eligibility criteria included histologically proven clinical stage II or III rectal cancer, main lesion located in the rectum with the lower margin below the peritoneal reflection, no clinical lateral lymph node enlargement: i.e. lateral lymph nodes with a short-axis diameter of <10 mm on computed tomography (CT) or magnetic resonance imaging (MRI), no clinical tumor invasion to adjacent organs (cT4b), Eastern Cooperative Oncology Group performance status (PS) 0 or 1, and age 20–75 years. Exclusion criteria were synchronous or metachronous (within 5 years) malignancies other than carcinoma in situ or mucosal carcinoma, pregnancy or breastfeeding in women, or a psychological disorder or severe mental illness. Patients undergoing treatment with systemic steroids, or with a history of myocardial infarction or unstable angina pectoris within 6 months, or with severe pulmonary emphysema or pulmonary fibrosis. The attending physician had the final decision for exclusion.

**Figure 1:**
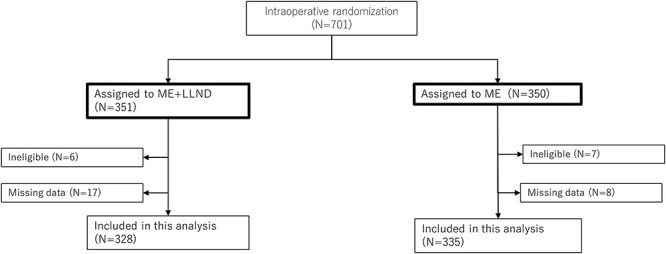
Consort diagram.

As shown in Figure 1, a total of 701 patients were randomized to the ME with LLND arm (*n* = 351) or the ME alone arm (*n* = 350). Of the 701 patients, excluding the 13 ineligible patients, 688 were eligible patients. Of the 688 eligible patients, 663 were selected for this study. Twenty-five patients who did not have sufficient data were excluded. Adjuvant chemotherapy, consisting of the Roswell Park regimen of 5-fluorouracil (500 g/m^2^) and L-leucovorin (250 mg/m^2^), was given to the pathological stage III patients. The present study aimed to identify clinical and pathological prognostic factors for RFS and OS using long-term (median 86 months) follow-up data from the JCOG0212 trial (4). 1 September 2017 was the data cut-off.

The factors for univariable analysis were selected based on clinical perspective. Continuous variables were divided into categories according to median or clinical point of view. As shown in Table 1, preoperative factors included age (≤60, ≥61 years), sex (male, female), performance status (PS) (0, 1), body mass index (≤25, 25–30, >30), tumor location (Ra: tumor center located above peritoneal reflection versus Rb and P: tumor center located below peritoneal reflection and anal canal), tumor circumferential location (anterior, lateral, posterior, circular), maximum tumor diameter (<5 cm, ≥5 cm), clinical T (cT2–3, cT4), distance of tumor from anal verge (<4 cm, 4–8 cm, ≥8 cm), short-axis diameter of lateral lymph node on CT (<3 mm, 3 to <5 mm, 5 to <7 mm, ≥7 mm), clinical N (cN0, cN1, cN2), clinical stage (II, III) and operative procedures such as abdominoperineal resection (APR) versus others and ME with LLND versus ME alone. Table 2 shows postoperative factors including histological grade (G1, G2, G3–4), pathological T (pT1–2, pT3, pT4a), anal distance from tumor to the resected line (<1 cm, ≥1 cm), number of metastatic lymph nodes (0, 1, 2–3, 4–6, ≥7), number of dissected lymph nodes (<12, ≥12) and pathological stage (I, II, III). The Japanese method was used to examine the radial margin macroscopically and microscopically (3).

**Table 1:** Patient characteristics and univariable analysis of preoperative prognostic factors for RFS and OS

Factors	5y RFS (95% CI)	*P*	5y OS (95% CI)	*P*
Age				
≤60 years (*n* = 309)	71.4 (66.0–76.1)		91.8 (88.1–94.4)	
≥61 years (*n* = 354)	76.8 (72.0–80.9)	0.701	92.1 (88.7–94.5)	0.415
Sex				
Male (*n* = 450)	72.4 (68.0–76.3)		90.4 (87.3–92.8)	
Female (*n* = 213)	78.3 (72.1–83.2)	0.083	95.3 (91.4–97.4)	0.036
Performance status				
0 (*n* = 628)	75.4 (71.8–78.6)		92.5 (90.1–94.3)	
1 (*n* = 35)	54.3 (36.6–69.0)	0.007	82.9 (65.8–91.9)	0.058
Body mass index				
≤25 (*n* = 504)	72.7 (68.6–76.4)		90.6 (87.7–92.9)	
25–30 (*n* = 145)	79.9 (72.4–85.6)	0.110	95.8 (91.0–98.1)	0.168
≥30 (*n* = 14)	71.4 (40.6–88.2)	0.715	100.0	0.287
**Tumor location**				
Ra (*n* = 193)	71.5 (64.5–77.3)		91.2 (86.2–94.4)	
Rb, *P* (*n* = 470)	75.4 (71.3–79.1)	0.303	92.3 (89.5–94.4)	0.726
**Tumor circumferential location**				
Anterior (*n* = 175)	73.0 (65.7–79.0)		92.5 (87.5–95.6)	
Lateral (*n* = 243)	75.6 (69.7–80.5)	0.567	92.1 (87.9–94.9)	0.949
Posterior (*n* = 185)	76.7 (69.9–82.1)	0.192	90.8 (85.6–94.2)	0.651
Circular (*n* = 60)	65.0 (51.5–75.6)	0.591	93.3 (83.2–97.4)	0.779
**Maximum tumor diameter**				
<5 cm (*n* = 318)	79.2 (74.3–83.3)		92.7 (89.3–95.1)	
≥5 cm (*n* = 345)	69.7 (64.5–74.3)	0.009	91.3 (87.7–93.8)	0.264
**Clinical T (cT)**				
cT2–3 (*n* = 643)	77.9 (73.2–81.8)		92.3 (90.0–94.2)	
cT4a (*n* = 20)	69.8 (64.2–74.7)	<0.001	79.4 (54.0–91.7)	0.101
**Distance of tumor from anal verge**				
<4 cm (*n* = 158)	74.6 (67.0–80.7)		91.7 (86.1–95.1)	
4–8 cm (*n* = 440)	75.4 (71.0–79.1)	0.811	92.0 (89.1–94.2)	0.882
≥8 cm (*n* = 65)	66.1 (53.2–76.2)	0.261	92.3 (82.4–96.7)	0.481
**Short-axis diameter of lateral lymph node**				
<3 mm (*n* = 493)	75.6 (71.5–79.1)		91.6 (88.8–93.8)	
3 to < 5 mm (*n* = 54)	77.8 (64.2–86.7)	0.815	94.4 (83.7–98.2)	0.568
5 to < 7 mm (*n* = 56)	67.8 (53.8–78.3)	0.281	94.5 (84.0–98.2)	0.472
≥7 mm (*n* = 29)	61.7 (41.6–76.7)	0.264	85.8 (66.5–94.4)	0.377
Missing (*n* = 31)	71.0 (51.6–83.7)	0.772	93.5 (76.6–98.3)	0.637
**Clinical N (cN)**				
cN0 (*n* = 369)	78.0 (73.4–81.9)		94.5 (91.7–96.4)	
cN1 (*n* = 249)	69.4 (63.2–74.7)	0.075	88.3 (83.6–91.7)	0.443
cN2 (*n* = 45)	70.8 (55.1–81.9)	0.246	91.1 (78.0–96.6)	0.306
**Clinical stage**				
II (*n* = 367)	77.9 (73.2–81.8)		94.5 (91.6–96.4)	
III (*n* = 296)	69.8 (64.2–74.7)	0.070	88.8 (84.6–91.9)	0.360
**Surgical procedures-1**				
APR (*n* = 112)	73.2 (63.9–80.4)		93.7 (87.3–97.0)	
Anterior resection or Hartmann (*n* = 551)	74.5 (70.6–77.9)	0.580	91.6 (88.9–93.6)	0.284
**Surgical procedures-2**				
ME with LLND (*n* = 328)	73.7 (68.6–78.1)		93.3 (90.0–95.5)	
ME alone (*n* = 335)	74.8 (69.8–79.1)	0.752	90.7 (87.0–93.3)	0.220

Ra, Tumor center located above the peritoneal reflection; Rb, Tumor center located below the peritoneal reflection; P, Anal canal; APR, abdominoperineal resection; ME, mesorectal excision; LLND, lateral lymph node dissection.

**Table 2:** Patient characteristics and univariable analysis of postoperative prognostic factors for RFS and OS

Factors	5y RFS (95% CI)	*P*	5y OS (95% CI)	*P*
**Histological grade**				
G1 (*n* = 205)	78.4 (72.1–83.4)		96.5 (92.9–98.3)	
G2 (*n* = 432)	72.8 (68.4–76.8)	0.040	90.0 (86.8–92.5)	0.014
G3–4 (*n* = 26)	65.4 (44.0–80.3)	0.042	88.5 (68.4–96.1)	0.012
**Pathological T (pT)**				
pT1–2 (*n* = 164)	92.6 (87.4–95.8)		98.2 (94.4–99.4)	
pT3 (*n* = 481)	69.3 (65.0–73.2)	<0.001	90.2 (87.1–92.5)	0.006
pT4a (*n* = 18)	38.9 (17.5–60.0)	<0.001	83.3 (56.8–94.3)	0.049
**Distance from tumor to the resected line**				
<1 cm (*n* = 31)	70.8 (51.4–83.6)		93.3 (75.8–98.3)	
≥1 cm (*n* = 632)	74.4 (70.8–77.7)	0.933	91.9 (89.5–93.8)	0.485
**No. of metastatic lymph nodes**				
0 (*n* = 368)	84.5 (80.3–87.8)		95.9 (93.3–97.5)	
1 (*n* = 83)	67.3 (56.0–76.3)	0.004	94.0 (86.1–97.4)	0.179
2–3 (*n* = 108)	70.4 (60.8–78.0)	0.011	90.7 (83.5–94.9)	0.125
4–6 (*n* = 58)	52.6 (38.9–64.6)	<0.001	86.2 (74.3–92.9)	0.006
≥7 (*n* = 46)	41.3 (27.1–54.9)	<0.001	67.4 (51.9–78.9)	<0.001
**No. of dissected lymph nodes**				
<12 (*n* = 47)	72.3 (57.2–82.9)		87.2 (73.8–94.1)	
≥12 (*n* = 616)	74.4 (70.8–77.7)	1.000	92.3 (89.9–94.2)	0.105
**Pathological stage**				
I (*n* = 125)	94.4 (88.5–97.3)		98.4 (93.7–99.6)	
II (*n* = 243)	79.4 (73.7–83.9)	0.006	94.6 (90.9–96.8)	0.459
III (*n* = 295)	61.5 (55.7–66.8)	<0.001	87.1 (82.7–90.4)	0.001

### Statistical analysis

**Figure 2:**
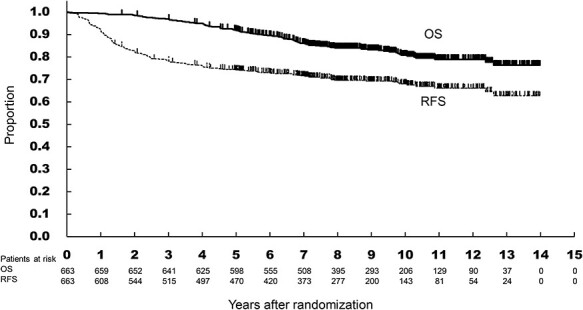
RFS and OS in 663 patients of this study.

Initially, Kaplan–Meier method was used to estimate time to event curves and 5-year RFS and OS with respective 95% confidence intervals (Fig. 2). Univariable Cox regression model was applied to investigate potential prognostic factors using preoperative factors and postoperative factors (Tables 1 and 2). Multivariable Cox regression analysis was performed in terms of RFS and OS using the preoperative and the combined preoperative and postoperative factors, respectively, and HR and the 95% confidence interval were estimated (Table 3). SAS®version 9.4 (SAS Institute, Cary, North Carolina, USA) was used to perform statistical analysis.

**Table 3:** Multivariable analysis of preoperative and postoperative factors for RFS and OS

		RFS				OS			
		Preoperative factors		Pre- and post-operative factors		Preoperative factors		Pre- and post-operative factors	
Factors	Level	HR (95% CI)	*P*	HR (95% CI)	*P*	HR (95% CI)	*P*	HR (95% CI)	*P*
Age	≤60 years	1		1		1		1	
	≥61 years	0.921 (0.690–1.227)	0.5729	0.931 (0.694–1.248)	0.6325	1.116 (0.763–1.631)	0.5716	1.182 (0.801–1.744)	0.3984
Sex	Female	1		1		1		1	
	Male	1.341 (0.979–1.836)	0.0676	1.326 (0.962–1.828)	0.0845	1.660 (1.073–2.568)	0.0228	1.537 (0.983–2.403)	0.0597
Performance status	0	1		1		1		1	
	1	2.079 (1.261–3.427)	0.0041	1.752 (1.047–2.931)	0.0327	1.878 (0.966–3.654)	0.0634	1.634 (0.824–3.239)	0.1596
Body mass index	≤25	1		1		1		1	
	25–30	0.759 (0.527–1.094)	0.1395	0.794 (0.549–1.147)	0.2191	0.706 (0.429–1.162)	0.1712	0.662 (0.398–1.102)	0.1128
	>30	1.236 (0.500–3.054)	0.6466	1.235 (0.492–3.103)	0.6528	1.617 (0.582–4.495)	0.3570	1.623 (0.559–4.714)	0.3732
Tumor location	Ra	1		1		1		1	
	Rb, P	0.983 (0.687–1.408)	0.9261	1.042 (0.719–1.510)	0.8278	1.039 (0.640–1.688)	0.8763	1.071 (0.650–1.766)	0.7871
Tumor circumferential location	Anterior	1		1		1		1	
	Lateral	1.001 (0.701–1.431)	0.9949	1.069 (0.741–1.541)	0.7223	1.061 (0.663–1.696)	0.8052	1.259 (0.773–2.050)	0.3543
	Posterior	0.820 (0.557–1.207)	0.3144	0.880 (0.593–1.307)	0.5276	0.924 (0.557–1.534)	0.7603	1.051 (0.621–1.776)	0.8538
	Circular	0.938 (0.558–1.576)	0.8080	0.766 (0.445–1.318)	0.3354	0.779 (0.371–1.637)	0.5103	0.803 (0.372–1.735)	0.5767
Maximum tumor diameter	<5 cm	1		1		1		1	
	≥5 cm	1.301 (0.950–1.781)	0.1012	1.212 (0.873–1.683)	0.2501	1.040 (0.688–1.572)	0.8536	1.104 (0.718–1.697)	0.6525
Clinical T (cT)	cT2–3	1		1		1		1	
	cT4a	2.718 (1.438–5.137)	0.0021	2.391 (1.215–4.704)	0.0116	2.486 (1.011–6.112)	0.0473	2.520 (0.936–6.783)	0.0674
Distance of tumor from anal verge	<4 cm	1		1		1		1	
	4–8 cm	1.111 (0.715–1.725)	0.6405	1.028 (0.635–1.665)	0.9103	1.067 (0.608–1.872)	0.8217	0.885 (0.465–1.684)	0.7095
	≥8 cm	1.271 (0.687–2.352)	0.4446	1.133 (0.585–2.197)	0.7108	0.789 (0.324–1.920)	0.6018	0.668 (0.250–1.785)	0.4208
Short-axis diameter of lateral lymph node	<3 mm	1		1		1		1	
	3–5 mm	0.850 (0.494–1.465)	0.5593	0.781 (0.449–1.358)	0.3804	0.715 (0.325–1.571)	0.4034	0.701 (0.312–1.575)	0.3893
	5–7 mm	1.341 (0.836–2.151)	0.2232	1.527 (0.936–2.489)	0.0898	1.256 (0.678–2.326)	0.4694	1.444 (0.766–2.723)	0.2557
	≥7 mm	1.421 (0.757–2.668)	0.2745	1.452 (0.773–2.728)	0.2459	1.579 (0.715–3.490)	0.2587	1.607 (0.725–3.563)	0.2429
	Unknown	0.921 (0.466–1.820)	0.8134	0.801 (0.401–1.600)	0.5291	0.780 (0.313–1.946)	0.5943	0.723 (0.286–1.832)	0.4945
Clinical N (cN)	cN0	1		1		1		1	
	cN1	1.231 (0.911–1.665)	0.1758	1.020 (0.745–1.397)	0.9022	1.251 (0.831–1.885)	0.2834	0.963 (0.626–1.482)	0.8653
	cN2	1.259 (0.718–2.205)	0.4214	0.792 (0.439–1.428)	0.4386	1.628 (0.790–3.353)	0.1866	0.799 (0.370–1.727)	0.5686
Surgical procedures 1	Anterior resection or Hartman	1		1		1		1	
	APR	1.258 (0.775–2.041)	0.3539	1.045 (0.610–1.789)	0.8729	1.361 (0.737–2.515)	0.3251	1.019 (0.502–2.066)	0.9589
Surgical procedure 2	ME+LLND	1		1		1		1	
	ME alone	1.013 (0.765–1.340)	0.9286	1.068 (0.797–1.432)	0.6580	1.200 (0.827–1.743)	0.3373	1.252 (0.842–1.862)	0.2672
Histological grade	G1			1				1	
	G2			1.273 (0.907–1.786)	0.1625			1.624 (1.003–2.631)	0.0487
	G3–4			1.554 (0.766–3.152)	0.2215			2.010 (0.839–4.818)	0.1173
Pathological T (pT)	pT1–2			1				1	
	pT3			2.122 (1.333–3.378)	0.0015			1.425 (0.806–2.520)	0.2233
	pT4a			3.478 (1.526–7.925)	0.0030			1.209 (0.371–3.937)	0.7533
Distance from tumor	<1 cm			1				1	
to the resected line	≧1 cm			0.814 (0.400–1.657)	0.5711			1.181 (0.408–3.418)	0.7588
No. of metastatic lymph nodes	0			1				1	
	1			1.678 (1.090–2.583)	0.0188			1.462 (0.787–2.714)	0.2291
	2–3			1.527 (1.011–2.307)	0.0443			1.506 (0.859–2.642)	0.1529
	4–6			2.716 (1.696–4.349)	0.0000			3.079 (1.613–5.878)	0.0007
	≥7			3.139 (1.906–5.170)	0.0000			6.719 (3.725–12.119)	0.0000
No. of dissected lymph nodes	<12			1				1	
	≥12			0.709 (0.389–1.293)	0.2619			0.503 (0.253–1.000)	0.0501

Ra, Tumor center located above the peritoneal reflection; Rb, Tumor center located below the peritoneal reflection; P: Anal canal; APR, abdominoperineal resection; ME, mesorectal excision; LLND, lateral lymph node dissection.

## Results

Figure 2 shows RFS and OS curves in all 663 patients of this study. The number of events for recurrence and death were 203 and 116, respectively. The 5-year RFS was 74.3% (95%CI, 70.7–77.4%) and the 5-year OS was 92.0% (95% CI, 89.6–93.8%). Tables 1 and 2 show the 5-year RFS and the 5-year OS in each category of the preoperative and postoperative prognostic factors. In Table 1, univariable analysis revealed that PS, maximum tumor diameter and clinical T (cT) were significant preoperative prognostic factors for RFS, and only sex for OS. In Table 2, histologic grade, pathological T (pT), number of metastatic lymph nodes and pathological stage were significant postoperative prognostic factors for RFS, and histological grade, pT, number of metastatic lymph nodes, and pathological stage for OS. Based on the univariable analysis, clinical stage and pathological stage were excluded from explanatory variable for multivariable analysis considering multicollinearity. As shown in Table 3, multivariable analysis revealed that PS and cT4a were preoperative independent risk factors for RFS, whereas those for OS were sex and cT4a. Particularly, cT4a was the common prognostic factor for RFS and OS and recorded the highest HRs (2.718, 2.486, respectively). Taking preoperative and postoperative risk factors into consideration, PS, cT4a, pT3, pT4a, and number of metastatic lymph nodes were significant prognostic factors for RFS, whereas histological G2 and number of metastatic lymph nodes were significant for OS. Among them, pT4a and ≥7 metastatic lymph nodes exhibited HR >3.0 for RFS and ≥4 metastatic lymph nodes showed HR > 3.0 for OS. In terms of diagnosis accuracy, sensitivity for pN positive was 59.3% (175/295) and specificity was 67.7% (249/368). As for the accuracy of depth of tumor invasion, the proportion of cT4a among pT4a was as low as 22.2% (4/18) and the proportion of cT2–3 among pT1–3 was 97.5% (629/645).

## Discussion

The JCOG0212 trial was a randomized controlled trial that compared mainly oncologic outcomes between the two surgical procedures (ME alone and ME with LLND) for stage II or III lower rectal cancer. To the best of our knowledge, this is the first paper to examine prognostic factors for lower rectal cancer in a phase III clinical trial without preoperative treatment in the TME (total ME) era (3,4).

The American Joint Committee on Cancer (AJCC) cancer staging manual on colon and rectum describes that T, N and M categories are essential for stage grouping (9). Tumor deposits are recommended for clinical care. Eight tumor-related prognostic factors are important to consider in making decisions about treatment: serum carcinoembryonic antigen (CEA) levels, tumor regression score, circumferential resection margin (CRM), lymphovascular invasion, perineural invasion, microsatellite instability, *KRAS* and *NRAS* mutation status and *BRAF* mutation. Age, sex, race, body mass index and PS are host-related prognostic factors. Some environmental prognostic factors such as treatment-related, education and quality of management also exist (10). In the present study for lower rectal cancer, univariable analysis showed that sex, PS, cT, maximum tumor diameter, histologic grade, pT, number of metastatic lymph nodes and pathological stage were significant prognostic factors (Tables 1 and 2), which almost supports those in the AJCC cancer staging manual (9). In addition, multivariable analysis revealed that cT4a was the common preoperative prognostic factor for RFS and OS with HR ≥2.4 and the number of metastatic lymph nodes showed the highest HRs of >3.0 among preoperative and postoperative prognostic factors for RFS and OS (Table 3). These results suggested T factor and N factor were the most established prognostic factors in patients with clinical stage II/III lower rectal cancer.

With the recent development of chemotherapy and radiotherapy, preoperative chemoradiation or total neoadjuvant treatment has become the most recommended choice for locally advanced lower rectal cancer in Western countries (11,12). Of note, RAPIDO trial introduced preoperative consolidation CAPOX/FOLFOX and improved disease-free survival (13) and PRODIGE23 trial introduced preoperative induction FOLFORINOX and improved disease-related treatment failure (14). Additional preoperative intensive chemotherapy to CRT and delayed surgery is highly expected to improve survival of rectal cancer. Clinical prognostic factors are now playing more crucial roles in preoperative treatment strategies. Survival data in this study indicated that patients with poor prognostic factors of cT4a, male gender and PS 1 should actively receive preoperative treatment if possible. Among preoperative prognostic factors, clinical T was one of the most powerful factors associated with RFS and OS. However, preoperative clinical diagnosis is less accurate than postoperative pathological diagnosis although there has been recent remarkable progress in imaging technology such as CT and MRI (15,16). Therefore, several factors should be taken consideration whether preoperative treatment is applied or not in each patient with cT4a. Moreover, it must be considered that postoperative pathologic staging following preoperative chemoradiotherapy underestimates the risk of developing distant metastases in rectal cancer compared with staging without preoperative chemoradiotherapy (17). Accordingly, careful follow-up and/or intensive postoperative treatment might be necessary even if down-staging was achieved for patients with cT4a.

There are several limitations in the present study. One is that CT data instead of MRI was used for preoperative clinical diagnosis. Yano et al. (15) reported that CT diagnosed lateral lymph node status with high accuracy (sensitivity 95%, specificity 94%). However, in our data preoperative CT diagnosed pathological lymph node status with low accuracy, which may explain the reason why not clinical N (cN) but pathological N (pN) was a prognostic factor. MRI has been considered to detect lateral pelvic lymph nodes more accurately than CT. It is also widely used for the preoperative assessment of risk factors, such as lower rectal plane including clinical CRM, venous invasion, depth of spread, nodal status, tumor height and tumor quadrant (including tumor deposits for lower rectal cancer) (16,18,19). Another limitation is that pathological CRM was not evaluated because pathological examination in Japan is different from that in Western countries and is performed after removal of the mesorectal fat for lymph node examination by surgeons (3). In the present study, microscopic radial resection margin—one of the prognostic factors (20)—was positively identified in only nine cases. The other prognostic factors of CEA, lymphovascular invasion, perineural invasion and genetic alterations (such as microsatellite instability, *KRAS*, *NRAS* and *BRAF*) (9) were also not prescribed on the protocol of the JCOG0212 trial. Lastly, the current standard adjuvant chemotherapy for rectal cancer without preoperative chemoradiotherapy is 5-fluorouracil with oxaliplatin worldwide. Therefore, the adjuvant chemotherapy of 5-fluorouracil and L-leucovorin for stage III patients in the present study is outdated.

In conclusion, the prognostic factors for lower rectal cancer without preoperative treatment were prospectively investigated in this large phase III study. Clinical stage II/III lower rectal cancer patients with cT4a should be a target of therapeutic development of neoadjuvant therapy. Postoperatively, intensive chemotherapy should be investigated for patients with more metastatic lymph nodes.
